# A Historical Analysis of Randomized Controlled Trials in the Management of Pain in Rotator Cuff Tears

**DOI:** 10.3390/jcm10184072

**Published:** 2021-09-09

**Authors:** Alessandra Berton, Umile Giuseppe Longo, Sergio De Salvatore, Gaia Sciotti, Giulia Santamaria, Ilaria Piergentili, Maria Grazia De Marinis, Vincenzo Denaro

**Affiliations:** 1 Department of Orthopaedic and Trauma Surgery, Campus Bio-Medico di Roma University, Via Alvaro del Portillo, 200, Trigoria, 00128 Rome, Italy; a.berton@unicampus.it (A.B.); s.desalvatore@unicampus.it (S.D.S.); ilaria.piergentili94@gmail.com (I.P.); denaro@unicampus.it (V.D.); 2 Research Unit Nursing Science, Campus Bio-Medico di Roma University, 00128 Rome, Italy; sciotti.cbm@gmail.com (G.S.); g.santamaria@unicampus.it (G.S.); m.demarinis@unicampus.it (M.G.D.M.)

**Keywords:** randomized controlled trial, rotator cuff tear, pain, modified Coleman methodology score, consolidated standards of reporting trials, quality

## Abstract

The aim of this analysis was to assess the quality of reporting of randomized controlled trials (RCTs) relating to pain management in rotator cuff (RC) tears. This review evaluated the quality of the studies in the literature regarding this topic through the use of some factors and trends. The online databases used to search all RCTs on the topic of RC surgery were Medline, Scopus, CINAHL, EMBASE, and CENTRAL. This research was completed in September 2020. To assess the quality of reports, the Consolidated Standards of Reporting Trials (CONSORT) and the modified Coleman methodology score (MCMS) were used. From the research, 262 articles emerged. Finally, 79 studies were included in this historical analysis. There were no statistically significant changes in MCMS across trials that included or did not include a CONSORT diagram (*p* = 0.10). A statistically significant difference in MCMS was discovered between papers produced prior to 2009 and publications produced after 2015 (*p* = 0.03). There was no association between the number of checklist items for each article and the Coleman score. During the years there has been a significant increase in both quantity and quality of RCTs relating to pain in RC tears.

## 1. Introduction

Rotator cuff tears are among the most prevalent cause of shoulder pain and disability for which patients need therapy [1,2]. With an incidence of approximately 6% and 30% in patients under and over 60 years [3]. Issues of the rotator cuff include alteration of collagen fiber structure, tenocytes, cellularity, and vascularity [4]. Surgical treatment is indicated in patients with persistent pain unresponsive to conservative treatment [1]. In the last ten years, the arthroscopic rotator cuff repairs (ARCR) overtook the open surgery in term of procedures performed [5,6]. ARCR is associated with shorter hospitalization time and complications, but it could be associated with postoperative pain [7,8].

So, during the postoperative period, the patient’s pain should be checked to reduce recovery times and to improve patient satisfaction [8]. It has also been verified that particular attention to pain enhances postoperative rehabilitation, helps early mobilization, and has better measures of functional recovery, including the range of motion and muscle power [9]. This type of pain is difficult to manage, so it is necessary to refer to studies that have performed well in terms of quality and reliability.

Randomized controlled trials (RCTs) are often considered the gold standard for a clinical trial. In this context, RCTs are useful to improve the health of patients affected by RC tears. In addition, randomization equipoises known and unknown prognostic elements among treatment groups [10].

These studies need to be carefully pursued, precisely because of the factors described above. Moreover, improving the allocation of randomization and performing an intent-to-treat analysis is useful to minimize the bias effects [11].

In recent years, CONSORT (Consolidated Standards of Reporting Trials) was introduced with the aim to improve the quality of RCTs reports, and to alleviate the problems arising from the inadequate reporting of these trials [12].

This paper aimed to evaluate the quality of reporting of all RCTs linked to pain treatments of RC tears.

## 2. Materials and Methods

A systematic review of the literature was performed following the preferred reported items for systematic review and meta-analysis statement (PRISMA). During the month of September 2020, the databases Medline, Scopus, CINAHL, EMBASE, and CENTRAL were searched. In order to find the RCTs in the literature on this topic, the “randomized controlled trials” filter and the following word combinations were used: “rotator cuff tear AND pain”; “rotator cuff repair AND pain”; “rotator cuff surgery AND pain”; “rotator cuff tear AND analgesia”; “rotator cuff repair AND analgesia”; and “rotator cuff surgery AND analgesia”. Initially, all articles were screened for relevance by title and abstract and obtaining the full-text article if the abstract did not allow the investigators to assess the defined inclusion and exclusion criteria. To obtain other relevant articles for the study we performed a cross-reference search of the selected articles. Inclusion criteria were: level of evidence I or II according to the Oxford Center of EBM; English language; studies on human patients; and focus on pain in patients affected by RC tears. Exclusion criteria were: pilot studies; commentary reports; preliminary studies; review articles; meta-analysis; animal and cadaveric studies; and conference papers. The title, abstracts, and full-text articles were screened independently by two researchers (G.S and C.D.N) that separately performed a careful reading of all trials. Furthermore, the CONSORT checklist was utilized individually by each investigator to evaluate each report. The CONSORT checklist consists of 25 items and 37 checklist items that focus on describing how the trial was conceived, analyzed, and interpreted. Each question receives a yes/no response based on the completeness of the information supplied in the survey. The flow diagram simply depicts the progression of all trial participants and is one of the elements on the CONSORT checklist [12].

### 2.1. Data Extraction

All extracted data were recorded using Microsoft Word and Excel. The characteristics of the study, such as the first author, year of publication, sample size, location of the study, level of evidence of the study, mean follow-up, the presence of CONSORT flow diagram, type of rehabilitation, and journal of publication were recorded. Other information of the studies, including the financial support or the number of centers involved in the treatment of patients, were abstracted.

### 2.2. Methodological Quality Assessment

The methodological quality of the included studies in this review was assessed independently using the modified Coleman methodology score (MCMS). Through this score and its eleven criteria, a score from 0 to 100 was calculated for each study. A score of 100 indicates an optimal study that avoids chance, various biases, and confounding factors [13]. The MCMS was used for the statistical analysis in order to evaluate correlation with other variables.

### 2.3. Assessment of Agreement

The information from the selected texts were extracted independently by two writers (G.S. and C.D.N.) and double-checked by a third author (G.F.). The two reviewers separately assessed the methodological quality and reliability of the findings using the MCMS.

### 2.4. Statistical Analysis

Descriptive statistics were used to present the results. The Shapiro–Wilk test for normal distribution of the data was used to assess variable normal distribution. To assess statistically significant differences of MCMS between the studies either containing the CONSORT diagram or not, the independent *t*-test was used. To evaluate the MCMS and the number of checklist items of the articles over the different years (2000–2009, 2010–2014, ≥2015) ANOVA and independent *t*-test were used. To statistically calculate significant differences of MCMS between dissimilar blinded studies (blinded patients, blinded observers, double-blinded and no-blinded), ANOVA and independent *t*-test were used. The inter-rater reliability in grading the Coleman score, as well as the Pearson’s correlation between the number of checklist items and the Coleman score for each article, were assessed. The inter-rater reliability of grading the CONSORT checklist was assessed using % agreement amongst raters.

## 3. Results

### 3.1. Study Characteristics

**Figure 1:**
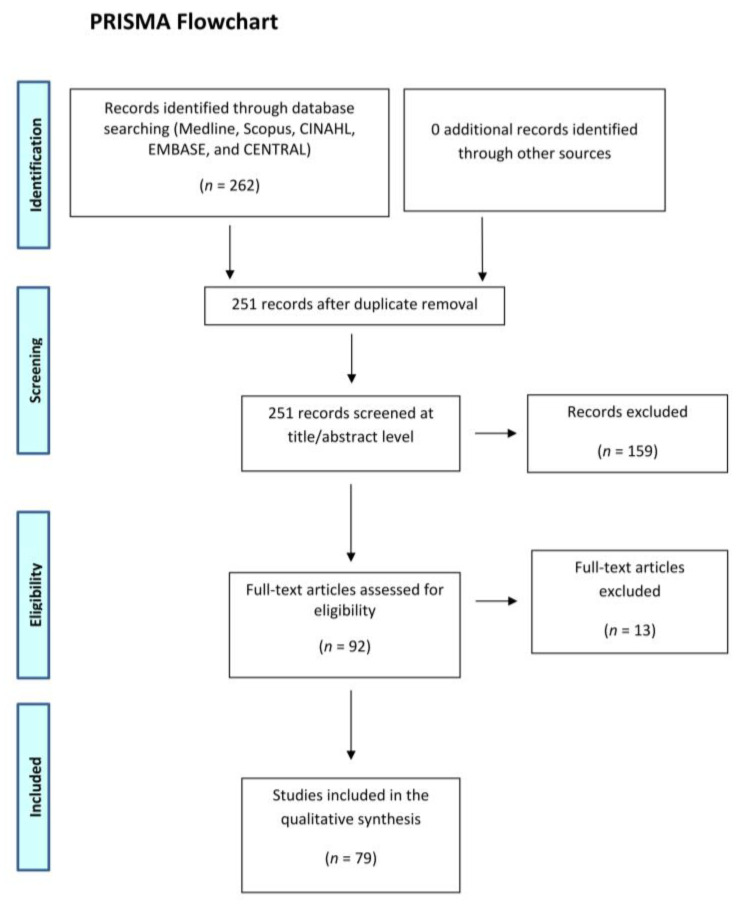
Preferred reporting items for systematic reviews and meta-analyses (PRISMA) 2009 flow diagram.

Figure 1 depicts the selecting procedure. A total of 251 articles were found throughout the literature search. Finally, the study comprised 79 trials.

A total of 5403 patients were initially analyzed in the involved studies. By the final follow-up, 212 patients were lost. A total of 74 of the 79 studies were single-centered [1,2,5,6,7,8,9,14,15,16,17,18,19,20,21,22,23,24,25,26,27,28,29,30,31,32,33,34,35,36,37,38,39,40,41,42,43,44,45,46,47,48,49,50,51,52,53,54,55,56,57,58,59,60,61,62,63,64,65,66,67,68,69,70,71,72,73,74,75,76,77,78,79,80] and only 1 report [81] was multi-centered. In the remaining 4 articles [82,83,84,85] it is unclear.

According to the Oxford Center of EBM, a total of 52 (66%) of 79 studies had a level of evidence I and 27 (34%) of 79 studies had a level of evidence II.

The analyzed studies were published in a total of 37 journals. Most of the included studies were published in the following four major journals: *J Shoulder Elbow Surg* (10 studies; 12.6%), *Arthroscopy* (9 studies; 11.3%), *The American Journal of Sports Medicine* (3 studies; 3.8% of the total), and *J Anesthesia and Analgesia* (5 studies; 6.3% of the total).

### 3.2. Topic

Despite the common general topic, 6 studies were focused on post-operative rehabilitation (3 on the immobilization with an abduction brace), 29 on the use of nerve block (in particular, 18 on the brachial plexus block, 9 on suprascapular nerve block, and 2 on supraclavicular block), 8 studies were focused on the intravenous patient-controlled analgesia, 4 on the intra-articular injection, and 11 on the subacromial injections. Finally, 7 studies were focused on the use and effect of specific drugs (gabapentin, zolpidem) and 1 study was focused on surgery. The remaining trials (13) were focused on other items linked to pain in patients undergoing rotator cuff surgery (miscellaneous).

### 3.3. Modified Coleman Methodology Score (MCMS)

The inter-rater reliability of the grading of Coleman score was 0.998. As for the quality indexes (the MCMS, the blinding of patients and observers, and the intent-to-treat) we considered in this review, there are considerations to be made. Of the 79 studies, only 11 included the intent-to-treat. Furthermore, 33 trials were double-blind, 23 were single-blind (11 blind patients and 12 blind observers), and the remaining 23 studies never performed the blinding of patients and observers.

The average MCMS in trials with blind patients was 65.09 (±7.39), the average MCMS in trials with blind observers was 67.67 (±7.33), the average MCMS in trials double-blinded was 69.06 (±7.50), and the average MCMS in trials without blinding was 63.17 (±6.84).

A statistically significant difference in MCMS between groups was found (*p* = 0.03). Multiple comparisons showed a statistically significant difference between trials double-blind and trials without blind (*p* = 0.02) (Table 1).

**Figure 2:**
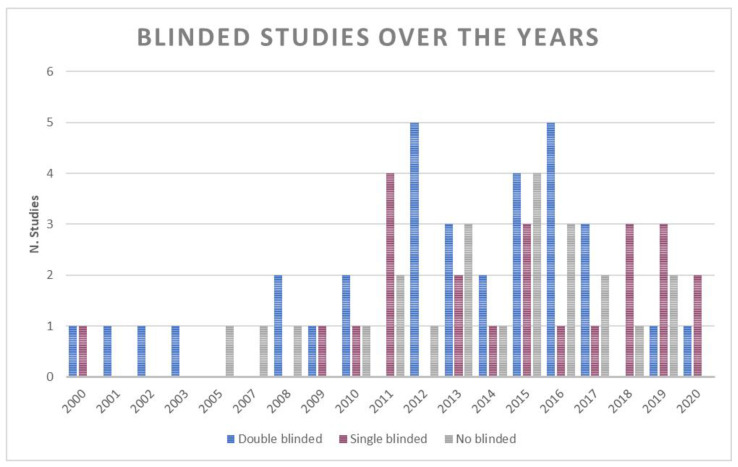
Number of double blinded, single blinded and no blinded studies over the years.

Figure 2 shows an increase in single blinded studies and a decrease in no-blinded and double-blinded studies after 2015.

Coleman score was greatest in articles featuring a CONSORT diagram. However, no statistically significant changes in MCMS were detected for trials that included or did not include a CONSORT diagram (*p* = 0.10) (Table 2).

### 3.4. Trends

**Figure 3:**
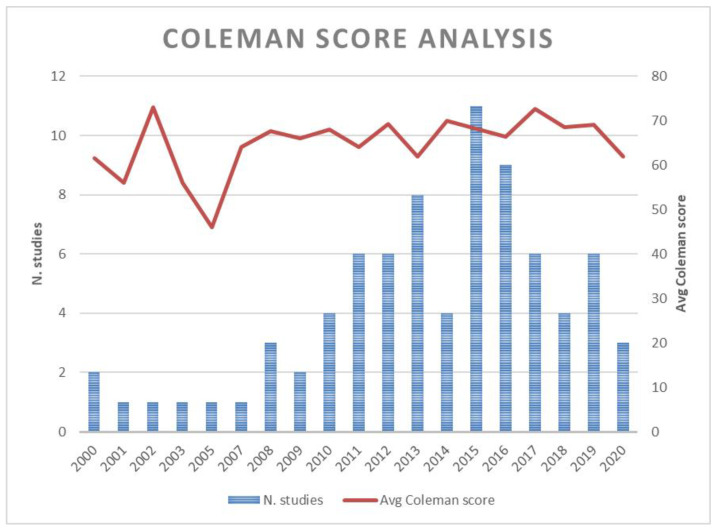
Number of studies and Coleman score analysis by years.

The earliest identified RCT was published in 2000. In the last 10 years (since 2010) 67 RCTs were published and 12 were published between 2000 and 2013. From about 2011, the number of RCTs published has increased compared to previous years. Above all, in 2015 more articles were published (Figure 3).

The average MCMS across all analyzed RCTs was 66.58 (±7.57). The average MCMS during the last five years registered a score of 68.18 (±7.36), between 2010 and 2014 the average MCMS was 66 (±7.13) and between 2000 and 2009 the average MCMS was 62.75 (±8.28). A mildly significant difference in MCMS between time was found (*p* = 0.08). No statistically significant differences in MCMS between articles written from 2000 to 2009 and articles written from 2010 to 2014 (*p* = 0.21) and between articles written from 2010 to 2014 and articles written after 2015 (*p* = 0.25) were found. A statistically significant difference in MCMS between articles written before 2009 and articles written after 2015 was found (*p* = 0.03) (Table 3).

### 3.5. Other Methodological Factors on the CONSORT Checklist

The inter-rater reliability of the CONSORT checklist grading was 0.985. Table S1 shows the rate of missing CONSORT checklist items for each trial, computed as the ratio of the number of checklist items with missing information to total checklist items for each trial.

No correlation (Pearson correlation = 0.11, *p* = 0.349) between the number of checklist items for each article and the corresponding Coleman score was found.

The average number of checklist items for each article across all analyzed RCTs was 20.96 (±3.44). The 12 studies completed until the year 2009 averaged 18.25 (±3.96) number of checklist items, the studies completed between 2010 and 2014 averaged 21 (±2.95) number of checklist items, and the 39 studies done after 2015 averaged 21.77 (±3.22) number of checklist items.

There was a statistically significant difference in the number of checklist items detected over time (*p* = 0.007). There were statistically significant variations in the amount of checklist items between publications produced between 2000 and 2009, papers published between 2010 and 2014 (*p* = 0.018), and publications produced after 2015 (*p* = 0.002). There was no statistically significant variation in the number of checklist items between articles produced between 2010 and 2014 and those produced after 2015 (*p* = 0.353) (Table 4).

Another important quality index is the presence of a CONSORT flow diagram that outlines the inclusion and exclusion of patients for the trial as well as the follow-up rate. Of the 79 studies, 34 (43% of total studies analyzed) included a CONSORT flow diagram. Among the studies completed between 2000 and 2014, 11 included this kind of diagram and the remaining 23 studies were completed after 2015. All the analyzed studies completed until 2009, do not include the CONSORT flow diagram. A statistically significant difference in the number of checklist items between studies with and without the CONSORT flow diagram was found (*p* < 0.001) (Table 5).

## 4. Discussion

A total of 79 RCTs with a focus on analgesia techniques and pain management were identified in the present review. The present review reported a progressive increase of RCTs during the last six years. This data could reflect a progressive interest from the international audience on rotator cuff disease. Only RCTs were deliberately analyzed in this review to assess their quality through the analysis of several factors. The collection of RCTs present in the literature on this topic allowed better visualization of the scientific evidence on pain in patients affected by rotator cuff tears. The importance of high-quality studies in the literature is evident. RCTs could influence the trend of a specific surgical procedure. For example, in 2009, two RCTs published in the USA changed the trend of vertebroplasty worldwide [86]. Therefore, providing high-quality evidence on rotator cuff disease could help surgeons improve their skills and patient outcomes.

Many authors adopted non-surgical strategies to reduce rotator cuff tears pain. In specific nerve block and subacromial injections were the most commonly adopted and effective techniques.

RCT is one of the common disorders of the musculoskeletal system in the ageing population [75,87,88]. In fact, this type of problem is linked to the worsening of the tendons, in many cases due to microtrauma or traumatic injuries [75,89,90]. Rotator cuff tear leads to medium-severe disability and, although there are other possible causes, RCT is commonly the cause of shoulder pain [39,91]. Several factors can contribute to an increased level of pain including female gender, early work resumption post-surgery, pre-existent capsulitis or bursitis, and additional performed subacromial decompression [5]. In the current scientific literature, the appropriate treatment for rotator cuff diseases is still a source of debate. However, in the case of patients with chronic and symptomatic rotator cuff tears the best treatment to reduce pain is surgical repair and there is increasing use of arthroscopic shoulder surgery [39]. Despite these improvements, rotator cuff surgery is related to a high incidence of postoperative pain [39,43]. Therefore, close control of postoperative pain is required to reduce the duration of hospitalization, to increase patient satisfaction, and clinical outcome [39,43]. Various treatment techniques have been developed in this regard and one of the most effective of these is regional nerve blocks [43]. Several studies have analyzed these techniques. The interscalene brachial plexus blocks (ISBs) lead to a reduction in postoperative pain even if for a short time. Suprascapular nerve blocks (SSNBs) are currently the most used method in this situation. While, a new kind of block has recently been introduced, often used in association with SSNB: it is axillary nerve block (ANB) [43].

Considering the Coleman score, there was an improvement in the quality of the studies completed in the last five years (average MCMS of 68.18) compared to the other studies published between 2000 and 2014. A statistically significant difference in MCMS between articles written before 2009 and articles written after 2015 was found. Unfortunately, the MCMS allows evaluation of only the quality of reporting, consequently, low scores are given to high-quality studies that are poorly written.

The CONSORT statement is an evidence-based minimum set of recommendations including a checklist and flow diagram for reporting RCTs to evaluate the quality of the studies [92]. In 2008, the CONSORT group developed an extension to the original statement that addressed methodological issues specific to trials of non-pharmacologic treatments (NPTs) [93]. The factors outlined in this extension included descriptions of how such trials should outline the calculation of sample sizes, the randomization procedure used, report the blinding status, and outline the flow of participants [93].

### 4.1. Limitations

The main limitation associated with the use of the modified Coleman methodology score (MCMS) as a quality measure of RCTs is that it assesses the quality of trial reporting rather than the quality of the trials themselves. In reality, stronger methodological protections may have been applied in certain studies that were not disclosed in the analysis. Furthermore, certain MCMS components might only be addressed in surgical studies (e.g., appropriate description of surgical technique and description of postoperative protocol). As a result, non-surgical investigations could never achieve the maximum score of 100.

**Figure 4:**
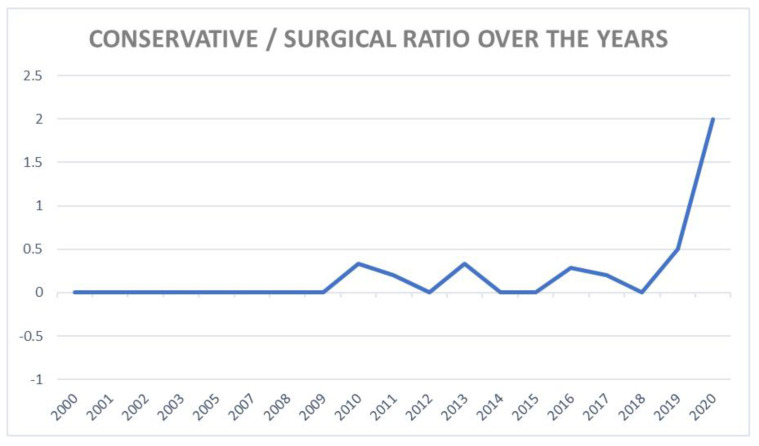
Ratio of conservative and surgical studies divided by years.

Therefore, the RCTs that involved surgery and that did not were assessed separately. Conservative studies increased after 2015, while surgical ones decreased. In fact, the conservative/surgical ratio is increasing since 2015 (Figure 4). Considering the years after 2015, the higher number of RCTs involving surgery could change the trend of MCMS.

This investigation only included English-language trials. Moreover, since too many variables between the studies included were present (type of treatment, sample size, follow up, baseline patient’s characteristics, initial tear size, etc.), it was not possible to perform a meta-analysis. We decided to only perform a systematic review, therefore it was not possible to test the heterogeneity between studies.

RCTs focusing on shoulder discomfort with intact rotator cuff tendons were also excluded.

### 4.2. Future Directions

Future studies might examine the impact that the pain in RCTs has had on clinical practices in RC surgery over time. Moreover, it would be important to assess the quality of reporting in other orthopedic areas to find the areas that require progress.

## 5. Conclusions

There has been a significant increase in the last years in both quantity and quality of RCT studies evaluating pain in RC tears. The number of RCT studying RC tears increased by 2010, with a peak in 2015. Concerning the quality of reporting of these RCTs has resulted in an improvement of the MCMS score registering a positive trend across the analyzed set. Papers written from 2015 had a higher average MCMS with respect to papers written between 2000 and 2009. Studies written from 2010 had a higher average number of checklist items with respect to studies written between 2000 and 2009. No correlation between the number of checklist items for each article and the respective Coleman score was found. The double-blinded articles showed higher Coleman scores with respect to the no blinded studies.

## Figures and Tables

**Table 1:** Mean MCMS between different blinded articles.

	Mean	SD ^1^	*N* ^2^	Blind Patients (*p*-Value)	Blind Observers (*p*-Value)	Double-Blinded (*p*-Value)
Blind patients	65.09	7.39	11	-	0.99	0.73
Blind observers	67.67	7.33	12	0.99	-	0.99
Double blinded	69.06	7.50	33	0.73	0.99	-
No blinded	63.17	6.84	23	0.99	0.52	0.02 *
Total	66.58	7.57	79			

^1^ Standard deviation, ^2^ number of studies, * statistically significant.

**Table 2:** Mean MCMS in articles with and without CONSORT flow-diagram.

CONSORT Flow-Diagram	Mean	SD ^1^	*N* ^2^	*p*-Value
YES	68.18	8.08	34	0.10
NO	65.38	7.02	45	

^1^ Standard deviation, ^2^ number of studies.

**Table 3:** Mean MCMS between different years groups.

Years	Mean	SD ^1^	*N* ^2^	2000–2009 (*p*-Value)	2010–2014 (*p*-Value)
2000–2009	62.75	8.28	12	-	0.21
2010–2014	66.00	7.13	28	0.21	-
2015+	68.18	7.36	39	0.03 *	0.25
Total	66.58	7.57	79		

^1^ Standard deviation, ^2^ number of studies, * statistically significant.

**Table 4:** Mean number of checklist items between different years groups.

Years	Mean	SD ^1^	*N* ^2^	2000–2009 (*p*-Value)	2010–2014 (*p*-Value)
2000–2009	18.25	3.96	12	-	0.018 *
2010–2014	21.00	2.95	26	0.018 *	-
2015+	21.77	3.22	39	0.002 *	0.353
Total	20.96	3.44	77		

^1^ Standard deviation, ^2^ number of studies, * statistically significant.

**Table 5:** Mean number of checklist items in articles with and without CONSORT flow-diagram.

CONSORT Flow-Diagram	Mean	SD ^1^	*N* ^2^	*p*-Value
YES	23.32	2.25	34	<0.001 *
NO	19.29	3.16	45	

^1^ Standard deviation, ^2^ number of studies, * statistically significant.

## Data Availability

The data presented in this study are available on request from the corresponding author.

## Supplementary Materials

The following are available online at https://www.mdpi.com/article/10.3390/jcm10184072/s1, Table S1: The rate of missed checklist items of CONSORT checklist for each trial.
