# Supplementary material for: A Historical Analysis of Randomized Controlled Trials in the Management of Pain in Rotator Cuff Tears
Source: J Clin Med. 2021 Sep 9;10(18):4072. doi: 10.3390/jcm10184072 (PMC8470804; doi:10.3390/jcm10184072)
Supplement: Supplementary file 1 [file jcm-10-04072-s001.zip › jcm-1328443-supplementary.pdf]

**Table S1. The rate of missed checklist items of CONSORT checklist for each trial**

| Articles | Year | No. Missed checklist item | Ratio missed checklist item |
|---|---|---|---|
| Alemanno | 2012 | 17 | 0.46 |
| Arias-Buria | 2015 | 13 | 0.35 |
| Banerjee | 2008 | 19 | 0.51 |
| Bang | 2010 | 17 | 0.46 |
| Barber | 2002 | 22 | 0.59 |
| Behr | 2012 | 18 | 0.49 |
| Borgeat | 2010 | 17 | 0.46 |
| Cabaton | 2019 | 10 | 0.27 |
| Cho CH | 2011 | 20 | 0.54 |
| Cho CH | 2015 | 20 | 0.54 |
| Cho NS | 2007 | 19 | 0.51 |
| Choi | 2018 | 21 | 0.57 |
| Choi EM | 2015 | 15 | 0.41 |
| Chou | 2010 | 14 | 0.38 |
| Ciccone | 2008 | 19 | 0.51 |
| Coghlan | 2009 | 8 | 0.22 |
| Conti | 2015 | 24 | 0.65 |
| Coory | 2019 | 11 | 0.30 |
| Culebras | 2001 | 19 | 0.51 |
| Delaunay | 2005 | 19 | 0.51 |
| Desmet | 2015 | 13 | 0.35 |
| Desroches | 2016 | 12 | 0.32 |
| Duzgun | 2011 | 19 | 0.51 |

| | | | |
|---|---|---|---|
| Faria-Silvia | 2014 | 21 | 0.57 |
| Forsythe | 2020 | 13 | 0.35 |
| Fredrickson | 2011 | 14 | 0.38 |
| Ghandour | 2019 | 14 | 0.38 |
| Gialanelli | 2011 | 18 | 0.49 |
| Gialanelli | 2013 | 18 | 0.49 |
| Gumina | 2012 | 15 | 0.41 |
| Gurger | 2019 | 15 | 0.41 |
| Han | 2013 | 17 | 0.46 |
| Hartrick | 2012 | 9 | 0.24 |
| Hofmann-Kiefer | 2008 | 16 | 0.43 |
| Hollman | 2017 | 14 | 0.38 |
| Hwang | 2020 | 12 | 0.32 |
| Ikemoto | 2015 | 19 | 0.51 |
| Jo CH | 2014 | 13 | 0.35 |
| Kasten | 2011 | 17 | 0.46 |
| Khashan | 2014 | 16 | 0.43 |
| Kim JH | 2018 | 18 | 0.49 |
| Kim JY | 2016 | 14 | 0.38 |
| Klein | 2000 | 19 | 0.51 |
| Ko SH | 2017 | 14 | 0.38 |
| Koh WU | 2016 | 13 | 0.35 |
| Kraeutler | 2015 | 18 | 0.49 |
| Lee HJ | 2015 | 16 | 0.43 |

| | | | |
|---|---|---|---|
| Lee JJ | 2014 | 13 | 0.35 |
| Lee JJ | 2015 | 13 | 0.35 |
| Lee JJ | 2017 | 11 | 0.30 |
| Liu XN | 2017 | 11 | 0.30 |
| Mahure | 2017 | 14 | 0.38 |
| Malik | 2016 | 17 | 0.46 |
| Merivirta | 2012 | 16 | 0.43 |
| Merivirta | 2013 | 14 | 0.38 |
| Merolla | 2015 | 14 | 0.38 |
| Oh CH | 2011 | 13 | 0.35 |
| Osti | 2015 | 15 | 0.41 |
| Park | 2016 | 17 | 0.46 |
| Perdreau | 2015 | 18 | 0.49 |
| Reynard | 2018 | 12 | 0.32 |
| Rha | 2013 | 12 | 0.32 |
| Salviz | 2013 | 12 | 0.32 |
| Schwartzberg | 2013 | 15 | 0.41 |
| Schwitzguebel | 2019 | 18 | 0.49 |
| Sethi | 2019 | 18 | 0.49 |
| Seven | 2017 | 17 | 0.46 |
| Shams | 2016 | 21 | 0.57 |
| Shin | 2013 | 17 | 0.46 |
| Syed | 2018 | 15 | 0.41 |
| Takada | 2009 | 22 | 0.59 |

| | | | |
|---|---|---|---|
| Tetzlaff | 2000 | 24 | 0.65 |
| Thackeray | 2013 | 20 | 0.54 |
| Turkmen | 2020 | 10 | 0.27 |
| Watanabe | 2016 | 17 | 0.46 |
| Wong | 2016 | 12 | 0.32 |
| Yamakado | 2014 | 18 | 0.49 |
| Yamamoto | 2003 | 19 | 0.51 |
| Yun | 2012 | 16 | 0.43 |
